# Kinematic Changes during Prolonged Fast-Walking in Old and Young Adults

**DOI:** 10.3389/fmed.2017.00207

**Published:** 2017-11-23

**Authors:** Camila Fonseca Oliveira, Edgar Ramos Vieira, Filipa Manuel Machado Sousa, João Paulo Vilas-Boas

**Affiliations:** ^1^Center of Research, Education, Innovation and Intervention in Sport (CIFI^2^D), Faculty of Sport, University of Porto, Porto, Portugal; ^2^Porto Biomechanics Laboratory (LABIOMEP), University of Porto, Porto, Portugal; ^3^Department of Physical Therapy, Florida International University, Miami, FL, United States

**Keywords:** active-aging, fast-walking, kinematics, falls prevention, intra-limb coordination

## Abstract

Walking biomechanics is known to be influenced by speed. However, most of the research examining the effects of walking speed and gait characteristics has been conducted after a fast-walking task, neglecting the changes that may occur during the task. The aim of the present study was to determine the impact of fast-walking over time on kinematics in young and old adults. Twenty-seven young adults (26.6 ± 6.0 years) and 23 old adults (71.0 ± 5.6 years) walked at 70% of their maximum heart rate for 20 min or until exhaustion, and the effects of fast-walking on temporospatial parameters and on angular kinematics were analyzed during the activity. During the protocol, both age-groups increased step-width variability. Significant effects of time were found for the ankle and hip at toe off for the older group. For the younger group, only the ankle angle at heel strike changed over time. For both groups, fast-walking induced changes in the coordination among the lower-limb angles that were more prominent during the swing phase of the gait. In conclusion, lower-limb kinematics changes in young adults were compatible with early signs of fatigue. The increased step-width variability in older adults may indicate an augmented risk of falling. Changes in the lower-limb walking kinematics of old adults suggest that the adjustments for weight acceptance and body propulsion were restricted to the hip and ankle joints. The kinematic changes among the lower-limb joint angles during the swing phase may compromise the quality of gait. These findings provide a foundation for future studies in the assessment of the risk of falls in older adults associated with walking at a faster pace.

## Introduction

The world population is aging. Approximately 14% of the European population are over the age of 65 and it is expected that this proportion will double by 2050 (1). With more than a third of older adults experiencing at least one fall per year, the consequences of falls represent a major problem for the health systems of many countries and represent huge associated costs (1). Falls often occur during walking (2) and can be a result of aging-related changes on gait (3). Gait changes observed in older adults are primarily due to reduced muscle strength and lower-limb joint range of motion as a result of physiological and neuromuscular changes (4–6). Aging-related changes in gait may be of particular concern when walking faster than usual. Increased walking speed may magnify the effects and limitations that may occur over time, and such assessments may highlight other factors that may be associated with falls. Irrespective of the factors underlying the age-related alterations on gait, several authors have associated fast-walking with an increased risk of falling (4, 7–9).

In general, healthy older adults are more susceptible to fall in outdoors activities (10), where walking at a faster pace can be a sporadic practice among this population. According to England and Granata (11), fast-walking may influence a successful locomotion by a combination of several mechanisms, as the ability to control movement could be disrupted by the effects of fast-walking on gait kinematics and other clinical correlates of stable walking. Studies involving fast-walking activity and its association with risk of falls are crucial for understanding the strategies that may be involved in falls’ prevention. However, most of the research examining the effects of fast-walking and its association with risk of falling (4, 7–9) were conducted after exposing the subjects to increased speed, neglecting changes in gait characteristics during the task. Thus, evaluating the effects of fast-walking activity over time on gait parameters may highlight potential threats that may arise while walking at a faster pace.

An individual’s gait strategy can be represented by their temporospatial parameters. Therefore, assessment of the gait parameters, namely fluctuations in temporospatial gait characteristics, may provide additional insight into the motor control of gait (12). In addition, as the coordination pattern between adjacent joint angles reflects a characteristic of the motor control of organizing adjacent structures in terms of timing and position to execute a movement (13), the intra-limb coordination was analyzed to display the impact of fast-walking on the coordinative synergism among the lower-limb angles. Thus, the present study was conducted to determine the impact of walking at a faster pace on temporospatial parameters and lower-limb joint kinematics in young and old adults during the activity.

## Materials and Methods

### Subjects

The participants were 27 young (26.6 ± 6.0 years) and 23 older adults (71.0 ± 5.6 years), the minimum age was 21 and 65 years for the groups of younger and older adults, respectively. Older participants were recruited from local community centers and younger adults from the local student population. Before the session, they were all screened to ensure eligibility. The exclusion criteria included any orthopedical, musculoskeletal, or cardiovascular constraints that might impair normal locomotion. All participants provided written informed consent using procedures approved by the Ethics Committee of the Faculty of Sport from the University of Porto, process number 10-2014.

### Procedures

Kinematic data were captured (100 Hz) using an 8-camera motion capture system (Qualisys, QTM). Reflective markers were placed on the anterior–posterior and posterior–superior iliac spines, bilaterally on the greater trochanter, medial and lateral femoral condyles, medial and lateral malleoli, end of the second toes, fifth metatarsal head, and calcanei. These markers were used to define the segments included in the biomechanical model (14). A standing calibration trial was recorded prior to the beginning of the task. Data were then processed using Visual 3D (C-motion, Rockville, MD, USA). Older participants wore a safety harness attached to the ceiling for safety.

### Protocol

All participants completed a familiarization session on a treadmill (AMTI Inc., MA, USA) walking at their preferred walking speed for 5 min. After that, the treadmill speed was increased by 0.5 km/h every 30 s until the participants reached the speed where they reached 70% of their age-predicted maximal heart rate (HR, 220 minus age in years). The participants were instructed to walk at this intensity for 20 min or until their self-assessed exhaustion. Two examiners were accompanying closely the participant during the task. HR was measured continuously during the test with a Polar HR monitor (2010 Polar Electro Oy, FI-90440 Kempele, Finland) to ensure subjects were walking at the required intensity. In addition, perceived exertion was assessed using a modified Borg 10-point scale (15). The relative speed was kept the same for each of the participants, and constant throughout the test. During the protocol, a variation of 10% of the target HR was permitted. The participants would be asked to stop the protocol if any of the following clinical indications were met: voluntary cessation, symptoms of cardiovascular discomfort, pulmonary discomfort, or the exertion reached 90% of their maximum HR, as recommended on ACSM’s guidelines for submaximal tests (16).

Dominant limb marker trajectories were collected during 30 s every minute until the end of the activity. The dominant limb was defined as the limb with which they would kick a football. Five equally distributed periods were later analyzed using Visual 3D (C-motion Inc., Rockville, MD, USA). The marker trajectories were filtered using a fourth-order low-pass Butterworth filter with a 12 Hz cut-off frequency. Gait cycle was defined by consecutive heel strikes of the dominant foot. Lower-limb angles were assigned with three rotational degrees of freedom and calculated using an XYZ Cardan sequence of rotations, which are equivalent to flexion/extension–abduction/adduction–axial rotation, respectively. Hip flexion, knee flexion, and dorsiflexion were displayed as positive displacement; whereas, hip extension, knee extension, and plantarflexion were displayed as a negative displacement.

The following parameters were assessed: sagittal angles of the hip, knee, and ankle at heel strike and toe off, cadence (number of steps per minute), stride length (distance between two successive right heel strikes), stride time (duration in seconds of a stride), step width (medio-lateral distance between heel position during heel strike of the left and right limbs). Sagittal joint angles at the ankle, knee and hip for each individual were time normalized to 100% of the gait cycle. Hip–knee–ankle sagittal angle plots were used as visual representatives of intra-limb joint coordination. The relative position between the adjacent joints was displayed along the gait cycle for the first and last stage of the protocol. Mean and SD of all kinematic data from approximately 30 strides were calculated on an individual level and were then averaged across participants for each group at every stage. Gait variability was assessed using SD for the angular kinematic data, and coefficient of variation $\text{CV}=\frac{\text{SD}}{\text{Mean}}\times 100\%$ for all temporospatial parameters.

### Data Analysis

Student’s *t*-test was used to identify between-group differences in fast-walking task-speed and demographics. Analyses of differences within- and between-group were assessed by repeated measures ANOVA. If the assumption of sphericity was failed, a Greenhouse–Geisser correction was used. All statistical procedures were performed using SPSS 25 (IBM, NY, USA) and a significance level of 0.05 was used.

## Results

Young and older adults achieved 70% at their maximum HR at significantly different velocities (*P* = 0.01). Old adults were not different from young adults in body mass but were shorter in height than the younger counterpart. Descriptive characteristics can be seen in Table 1.

**Table 1:** Characteristics of study’s participants.

Characteristics	Young adults (*n* = 27)	Old adults (*n* = 23)
Age (years)*	26.6 ± 6.0	71.1 ± 5.6
Height (m)*	1.72 ± 0.11	1.63 ± 0.10
Body mass (kg)	68.0 ± 15.5	69.9 ± 10.2
Fast-walking speed (m/s)*	1.91 ± 0.20	1.22 ± 0.31

*Values are displayed as mean ± SD*.

*Significant difference (*p* < 0.05) between age-groups using independent sample *t*-tests.

**Figure 1:**
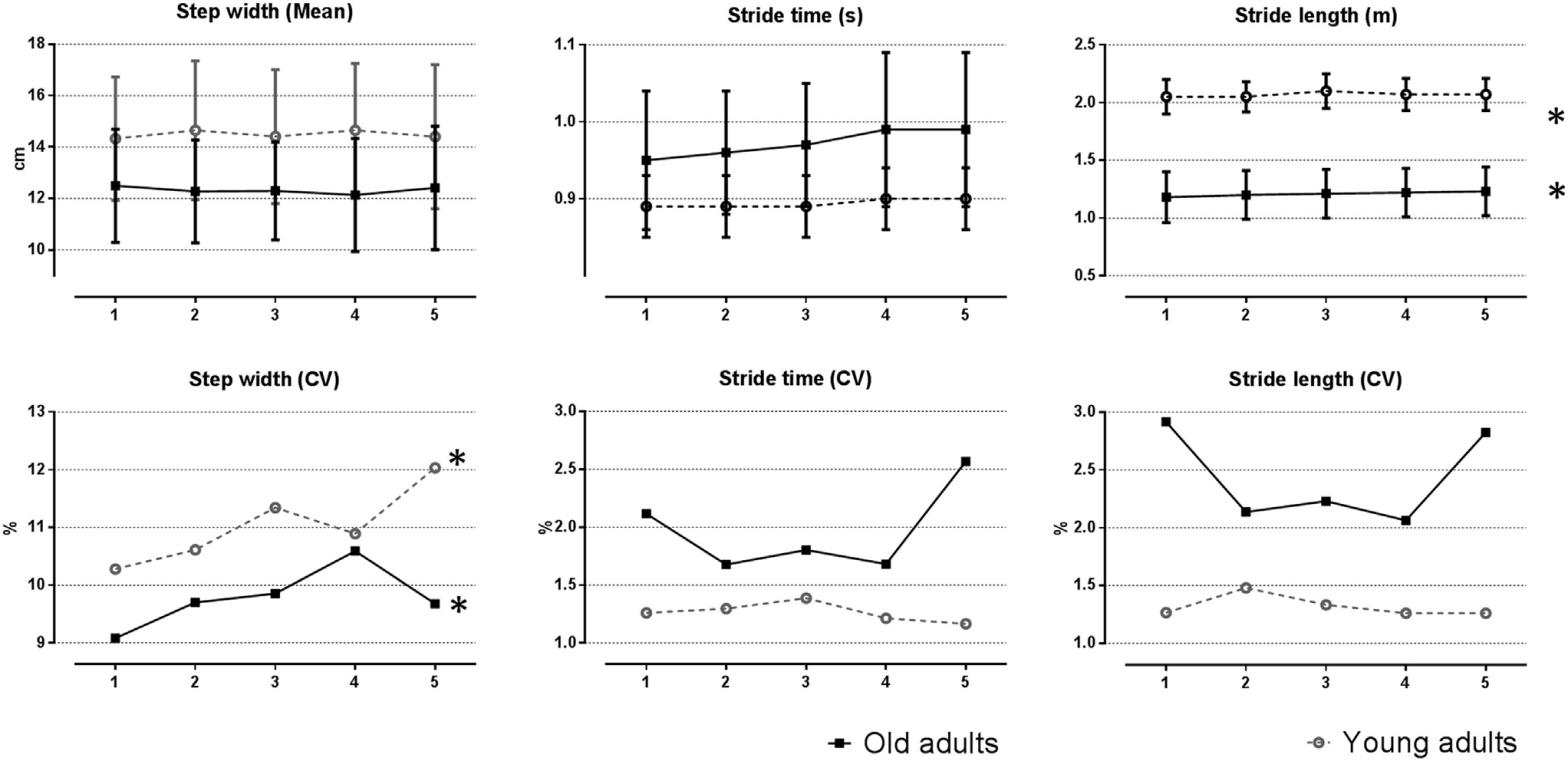
Temporospatial parameters during fast-walking activity at the top. The mean and SD for each subject were subsequently averaged across subjects within the age-group. At the bottom, differences in the coefficient of variation revealed the differences in temporospatial variability along the task. *Indicates statistically significant time effect.

Since gait strategies are directly influenced by the walking speed, both groups had different cadence, stride length, stride time, and step-width mean values. During the activity, both age-groups showed slight, but significant increase of stride length. Young and old adults kept the mean values of the step-width throughout the protocol. The stride time increased only for the group of older adults. Levels of variability (assessed by the coefficient of variation) were higher in the elderly group for the stride length and the stride time (see Figure 1). In both age-groups, statistically significant main effects of time were found only for the step-width coefficient of variation (*P* = 0.03).

**Figure 2:**
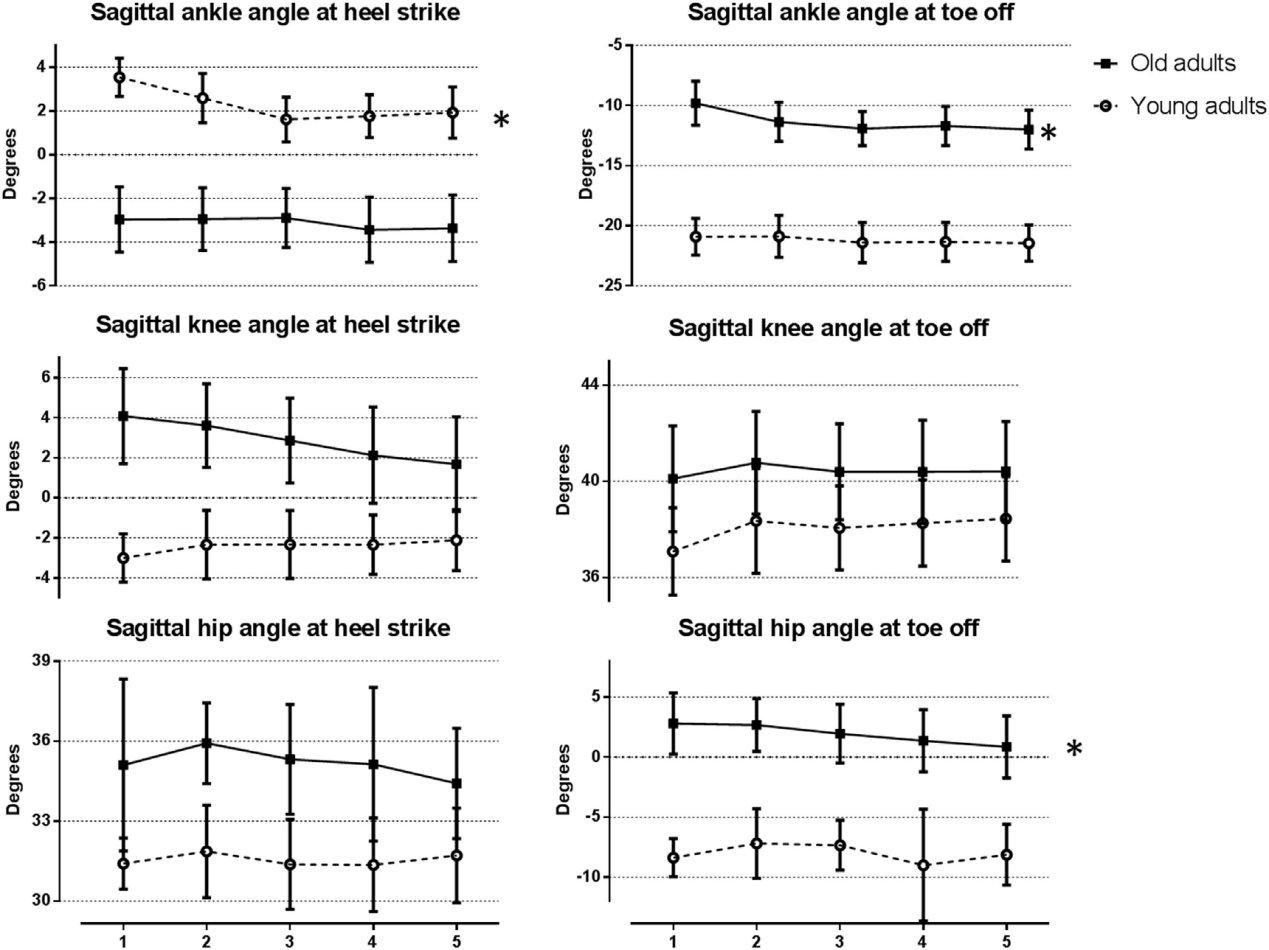
Angular displacements for the hip, knee, and ankle at heel strike and toe off in young and old adults during the five time points along the fast-walking activity. *Indicates statistically significant time effect. The mean and SD for each subject were subsequently averaged across subjects within the age-group.

Between-group comparisons of joint angular kinematics showed differences for the mean values for the ankle, knee (at heel strike), and hip (at toe off). Significant effects of time were found for the ankle and hip at toe off for the older group. For the younger group, only the ankle angle at heel strike changed over time. Older adults had higher lower-limb angular variability at heel strike for the ankle and knee than the younger adults. No time effects were found for the lower-limb joint angle’s variability. Time and group differences of the sagittal angles at the heel strike and toe off are presented in Table 2, and can be seen in Figure 2.

**Table 2:** Means and SDs for gait variables across all the time points during fast-walking activity.

Variable	Group	Time points					*P* ANOVA		
		T1	T2	T3	T4	T5	Group	Time	Interaction
**Temporospatial parameters**									

Cadence	OA	**61.4 ± 0.8**	**62.2 ± 0.9**	**61.3 ± 0.8**	**60.2 ± 0.9**	**58.2 ± 0.9**	**0.001**	**0.004**	0.518
	YA	**65.4 ± 0.8**	**63.8 ± 0.9**	**63.2 ± 0.8**	**62.2 ± 0.8**	**61.2 ± 0.7**			

Stride time (s)	OA	**0.95 ± 0.09**	**0.96 ± 0.08**	**0.97 ± 0.08**	**0.99 ± 0.10**	**0.99 ± 0.10**	**0.022**	**0.001**	**0.020**
	YA	0.89 ± 0.04	0.89 ± 0.04	0.89 ± 0.04	0.90 ± 0.04	0.90 ± 0.04			

Step width (cm)	OA	12.5 ± 2.2	12.2 ± 2.0	12.2 ± 1.9	12.1 ± 2.2	12.2 ± 2.4	**0.002**	0.613	0.226
	YA	14.3 ± 2.4	14.6 ± 2.7	14.4 ± 2.6	14.6 ± 2.6	14.1 ± 2.8			

Stride length (m)	OA	**1.18 ± 0.22**	**1.2 ± 0.21**	**1.21 ± 0.21**	**1.22 ± 0.21**	**1.23 ± 0.21**	**0.001**	**0.001**	0.172
	YA	**2.05 ± 0.15**	**2.05 ± 0.13**	**2.1 ± 0.15**	**2.07 ± 14**	**2.07 ± 0.14**			

Stride length (CV)	OA	2.9 ± 2.6	2.1 ± 1.3	2.2 ± 1.3	2.1 ± 1.2	2.9 ± 3.8	**0.012**	0.341	0.174
	YA	1.3 ± 0.4	1.5 ± 1.0	1.3 ± 0.7	1.3 ± 0.3	1.3 ± 0.3			

Stride time (CV)	OA	2.1 ± 1.8	1.7 ± 0.8	1.8 ± 1.2	1.7 ± 0.9	2.5 ± 1.4	**0.007**	0.377	0.178
	YA	1.2 ± 0.4	1.3 ± 0.5	1.4 ± 1.0	1.2 ± 0.4	1.2 ± 0.3			

Step width (CV)	OA	9.6 ± 3.2	10.1 ± 3.6	10.4 ± 2.9	11.2 ± 3.8	11.6 ± 7.9	0.319	**0.031**	0.402
	YA	11.5 ± 8.0	11.9 ± 8.7	12.6 ± 8.9	12.2 ± 8.5	13.2 ± 10.2			

**Angular kinematics**									

	**Differences in the mean values along the activity**								

Ankle angular position at HS	OA	−2.9 ± 5.5	−2.9 ± 5.0	−2.9 ± 4.6	−3.4 ± 4.8	−3.4 ± 5.0	**0.001**	**0.032**	**0.034**
	YA	**3.5 ± 4.0**	**2.6 ± 5.0**	**1.3 ± 4.6**	**1.7 ± 4.0**	**1.9 ± 4.2**			

Ankle angular position at TO	OA	**−9.8 ± 6.0**	**−11.4 ± 6.4**	**−11.9 ± 6.4**	**−11.8 ± 7.0**	**−12 ± 6.5**	**0.001**	**0.021**	**0.041**
	YA	−20.9 ± 10	−20.1 ± 11	−21.4 ± 10	−21.3 ± 10	−21.4 ± 10			

Knee angular position at HS	OA	4.0 ± 6.0	3.6 ± 6.6	2.9 ± 6.9	2.2 ± 6.5	1.7 ± 7.0	**0.001**	0.126	0.101
	YA	−3.0 ± 3.4	−2.3 ± 3.4	−2.3 ± 3.2	−2.3 ± 3.4	−2.1 ± 3.2			

Knee angular position at TO	OA	40.1 ± 6.7	40.8 ± 6.4	40.4 ± 6.5	40.4 ± 7.1	40.4 ± 7.2	0.127	0.548	0.374
	YA	37.1 ± 4.0	38.4 ± 4.1	38.0 ± 4.5	38.3 ± 4.4	38.4 ± 4.1			

Hip angle position at HS	OA	35. 1 ± 7.6	35.9 ± 9.3	35.3 ± 9.4	35.1 ± 8.9	32.9 ± 10.8	0.103	0.279	0.246
	YA	31.4 ± 5.8	31.9 ± 6.2	31.4 ± 6.6	31.4 ± 6.0	31.7 ± 6.1			

Hip angle position at TO	OA	**2.8 ± 9.9**	**2.7 ± 9.5**	**1.9 ± 9.8**	**1.4 ± 9.1**	**0.8 ± 9.4**	**0.00**	**0.017**	0.032
	YA	−8.4 ± 6.8	−7.1 ± 7.3	−7.4 ± 6.9	−9.0 ± 7.1	−8.1 ± 6.4			

	**Differences in the SD along the activity**								

Ankle angular position at HS	OA	1.5 ± 0.8	1.4 ± 0.7	1.3 ± 0.9	1.5 ± 1.2	1.5 ± 1.2	**0.013**	0.657	0.706
	YA	0.9 ± 03	1.1 ± 0.9	1.0 ± 0.4	1.0 ± 0.4	1.2 ± 0.7			

Ankle angular position at TO	OA	1.8 ± 0.9	1.6 ± 0.8	1.4 ± 0.7	1.6 = ± 0.7	1.6 ± 0.8	0.946	0.562	0.089
	YA	1.5 ± 0.5	1.7 ± 0.8	1.7 ± 0.6	1.6 ± 0.9	1.5 ± 0.6			

Knee angular position at HS	OA	2.4 ± 1.3	2.1 ± 1.1	2.1 ± 1.3	2.4 ± 2.3	2.4 ± 2.0	**0.012**	0.939	0.326
	YA	1.2 ± 0.7	1.7 ± 1.2	1.7 ± 1.2	1.5 ± 1.0	1.5 ± 1.0			

Knee angular position at TO	OA	2.1 ± 0.7	2.1 ± 1.1	2 ± 1.1	2.1 ± 1.0	2.1 = 1.1	0.159	0.533	0.684
	YA	1.8 ± 0.7	2.2 ± 2.0	1.7 ± 0.6	1.8 ± 0.9	1.7 ± 0.6			

Hip angle position at HS	OA	3.2 ± 3.8	1.5 ± 0.9	2.0 ± 1.5	2.9 ± 3.4	2.0 ± 2.8	0.078	0.303	**0.005**
	YA	0.9 ± 0.3	1.7 ± 1.9	1.7 ± 1.1	1.8 ± 1.1	1.8 ± 1.4			

Hip angle position at TO	OA	2.5 ± 1.6	2.2 ± 1.4	2.4 ± 2.0	2.6 ± 2.1	2.5 ± 2.4	0.667	0.344	0.366
	YA	1.6 ± 0.8	2.9 ± 4.4	2.1 ± 1.5	2.0 ± 2.1	2.5 ± 2.1			

*Values are presented as mean ± SD, bold indicates p < 0.05. OA, old adults; YA, young adults; CV, coefficient of variation; HS, heel strike; TO, toe off*.

**Figure 3:**
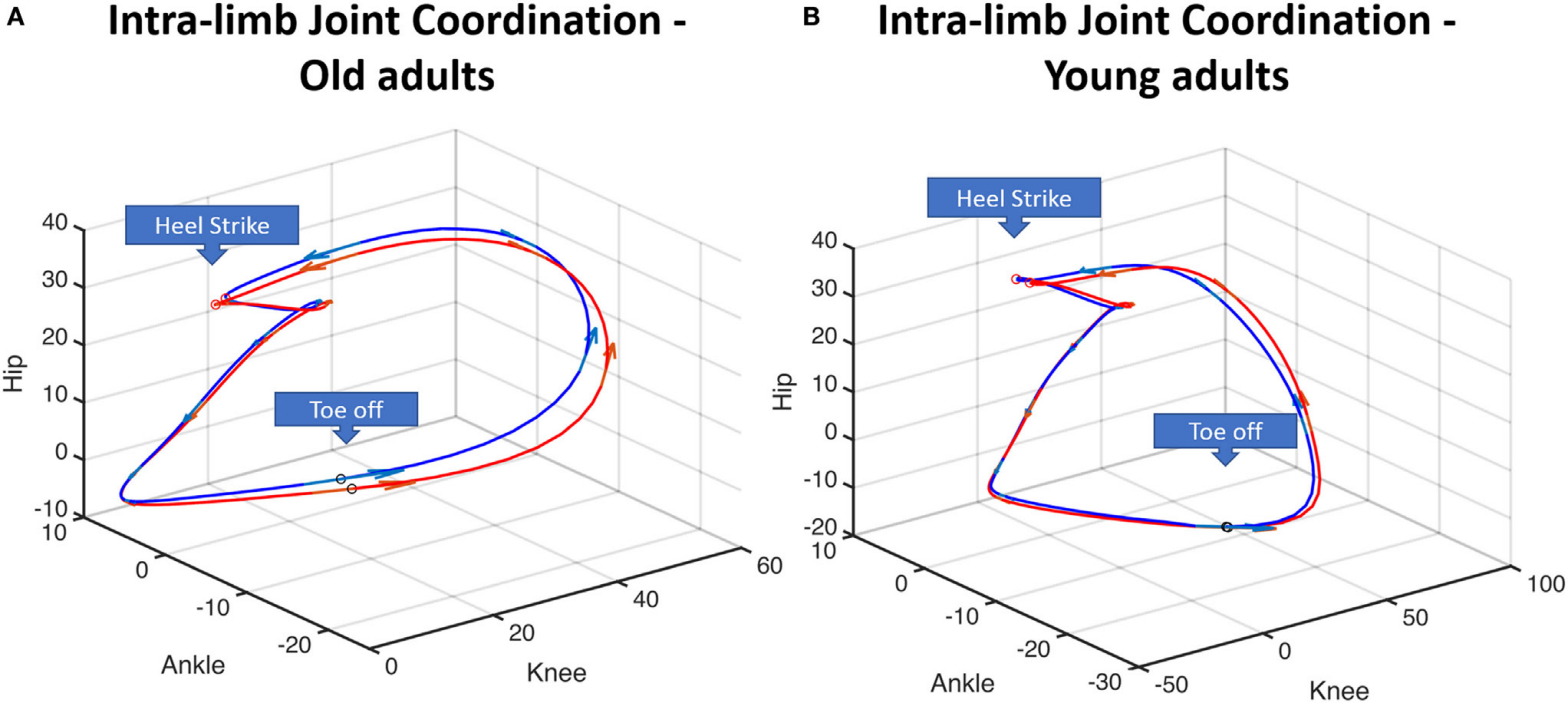
Illustration of hip angle–ankle angle–knee angle intra-limb coordination patterns for the old adults **(A)** and the young adults **(B)**. Blue line represents the relative hip-knee-ankle position averaged for the group for the first stage; red line represents the relative hip-knee-ankle position averaged for the group for the fifth/last stage. These two stages are displayed to highlight the differences induced by the activity. Arrows indicate the direction of the gait.

The intra-limb trajectories representing the coordination among adjacent angles were displayed for the first (blue line) and last stage (red line) of the protocol, as can be seen in Figure 3. For both age-groups, visual inspection of the trajectories revealed that the differences between the intra-limb trajectories were more evident during the swing phase, especially for the old group.

## Discussion

The objective of this study was to explore the continuous acute effect of a sustained fast-walking along the activity on the gait parameters in healthy young and old adults. We searched for supplementary information regarding potential risks induced by a more demanding activity than walking at a freely chosen speed as in daily life activities.

Along the activity, both age-groups changed their gait strategy by having fewer, but longer strides. Even though such strategy may minimize the energy cost of walking (17), it may also increase the risk of injury at the knee (18). Stride length and stride time variability were greater in old adults when compared to young participants herein. Similar findings were reported by Kang and Dingwell (19). Literature regarding age-related differences in gait variability (defined here as fluctuations in temporospatial gait characteristics) and its relationship with walking speed are contradictory. Because older adults typically walk slower, increased gait variability in healthy older adults was considered to be related to their slow walking speed. However, previous studies have suggested that age-related changes in variability rather than be a manifestation of walking speed is more likely to reflect an underlying impairment of the motor system (19–21). With respect to changes in temporospatial variability, the most relevant finding here was the effect of fast-walking on step-width variability in both age-groups. Increased step-width variability has been suggested to be a necessary adaptation to maintain lateral stability. The control of lateral foot displacement is regulated by somatosensory, visual, and vestibular input (22). Such an increase in step-width variability may be explained by the reduction of sensory information induced by the activity (23), which is congruent with the negative effects found on postural control and balance induced by a similar walking protocol of previous studies (24, 25). Thus, during fast-walking, for both groups, the increased step-width variability could be a signal of incipient lateral instability, and loss of balance (26, 27). In addition, previous studies have associated the increment in step-width variability to increased risk of falls in old adults (2, 28).

Fast-walking resulted in significant changes on the lower-limb kinematics of both age-groups. Toward the end of the practice, older adults were initiating the swing phase with additional ankle plantarflexion, and a less extended hip while no changes were observed at the knee. Thus, the necessary adjustments that may have taken place for weight acceptance and body propulsion during the activity were restricted to the hip and ankle joints in old adults. A reduction in hip extension may imply a functional tightness that would be preventing the hip to fully extend at the toe-off (29). The increased ankle plantarflexion seems to be a compensatory mechanism to the reduction in hip extension since they managed to keep and even increase the stride length (30). These findings suggest that the relative contribution of the ankle plantarflexion for propelling the body forward has increased during the activity. Meanwhile, young adults manifested early signs of muscle fatigue, as we can see through their progressive reduction of the ankle dorsiflexion at landing (31). Old adults displayed higher variability for the ankle and knee angular position at heel contact, but levels of kinematic variability did not change throughout the protocol. However, in the present study, analyses regarding variability of angular kinematics were restricted to two events of the gait cycle. Further study on progressive effects of fast-walking activity should assess kinematical variability throughout the gait cycle to provide a complete understanding of the effects of fast-walking.

As we could observe from the intra-limb coordination trajectories, both groups displayed different patterns along the gait cycle. In interpreting this finding, one must consider that this difference may be associated with differences in the walking speed between groups and not exclusively to the age-related differences in walking pattern. The intra-limb joint coordination reflects a characteristic of the motor control of organizing adjacent structures in terms of timing and position to execute a movement (13, 32–34). Several studies have studied this outcome to obtain complementary information into gait control across different neuromuscular disorders (32, 34, 35). Changes in the state of intra-limb joint coordination may emerge due to alteration on any component of the motor system, as those caused by fatigue (33), or yet, alterations on muscle co-activation (34). The shift between the first and the fifth (last) intra-limb coordination trajectories reflect the effect of the activity on the related positions among the joint angles throughout the gait cycle and was more evident during the swing phase. Differences in the coordination among the lower-limb adjacent joints may influence the swing foot trajectory (36), which, in turn, may affect gait parameters related to the risk of falls by tripping and slipping.

Some limitations of this study were the absence of kinetic analyses and the use of a treadmill, which may decrease gait variability (37). However, the use of a treadmill allowed us to analyze continuous gait cycles. To the best of our knowledge, this study was the first to describe alterations on gait during sustained faster pace walking. Our findings revealed that old adults progressively changed their kinematics at hip and ankle during the task. Meanwhile, young adults showed incipient signs of fatigue at the ankle joint. Both age-groups changed their gait strategy by reducing cadence and increasing stride length and increased the variability of step-width. Walking at faster pace had no effect on angular kinematics variability assessed at the heel strike and toe off. However, further analyses should verify the effect of fast-walking throughout the gait cycle. In addition, the differences found in intra-limb coordination trajectories revealed that changes were more prominent during the swing phase. Which, in turn, reveals that the changes were phase dependent within the gait cycle. When walking at their typical pace, healthy active elderly subjects with increased step-width variability have more chances to fall (2, 28). Lower-limb kinematics during walking are strictly associated with the safe performance of two important events during the swing phase of gait, the minimum toe clearance, and the heel contact velocity (36, 38). Both gait events are directly linked to the risk of falls by tripping, and slipping, respectively (3, 36). Therefore, the changes induced after a certain period walking faster than usual, shown here, may compromise the quality of gait increasing the risk of falls. Future work should investigate kinematic changes throughout the gait cycle, particularly at the swing phase. The findings reported herein are important for addressing potential risks associated with walking at a faster pace.

## Ethics Statement

All subjects gave written informed consent in accordance with the Declaration of Helsinki. The protocol was approved by the Ethics Committee of the Faculty of Sport from the University of Porto, process number 10-2014.

## Author Contributions

CO, principal author, is responsible for the concept, design and intellectual content of the work, data process analysis, and interpretation. EV contributed with the intellectual content of the work, drafting ideas and reviewed analysis, and the final version. FS contributed with the data acquisition, drafting the work, and final revision. JV-B substantially contributed to the analysis and interpretation of the data and critically improved the content of the work. All the authors approved the final version of the manuscript, and declare to have no conflict of interest associated with this work, as well declare to be accountable for all aspects of it.

## Conflict of Interest Statement

The authors declare that the research was conducted in the absence of any commercial or financial relationships that could be construed as a potential conflict of interest.
